# hUC-MSC Combined with DHEA Alleviates Ovarian Senescence in Naturally Aging Mice through Enhancing Antioxidant Capacity and Inhibiting Inflammatory Response

**DOI:** 10.1155/2024/3100942

**Published:** 2024-07-30

**Authors:** Chun-Yi Guan, Dan Zhang, Xue-Cheng Sun, Xu Ma, Hong-Fei Xia

**Affiliations:** ^1^ Reproductive and Genetic Center and NHC Key Laboratory of Reproductive Health Engineering Technology Research National Research Institute for Family Planning (NRIFP), Beijing 100081, China; ^2^ Graduate School Peking Union Medical College, Beijing 100005, China

## Abstract

The ovary is an important organ for women to maintain reproductive and endocrine functions. Ovarian aging can lead to female reproductive aging, which is a key factor causing rapid aging of the female body. Umbilical cord-derived MSCs (UC-MSCs) play a therapeutic role in various degenerative diseases. Dehydroepiandrosterone (DHEA) is widely used in the treatment of reversing oocyte quality. However, it is unclear whether UC-MSCs combined with DHEA supplementation can improve ovarian senescence in naturally aging mice. To address this question, we studied the influence of the combination of human UC-MSCs (hUC-MSCs) and DHEA on ovarian morphology and function in naturally aging mice. The results showed a significant augmentation in the number of primary follicles, as well as a significant upregulation of estradiol (E2), follicle-stimulating hormone (FSH), and anti-Mullerian hormone (AMH) hormone levels, and a significant increase in survival rate in naturally aging mice treated by hUC-MSCs and DHEA. Moreover, the combination of hUC-MSCs and DHEA significantly reduced the reactive oxygen species (ROS) level and downregulated the expression levels of proinflammatory factors IL-6, IL-18, and TNF-*α*. Furthermore, the PI3K/AKT/mTOR pathway was inhibited. Conclusively, the combination therapy of hUC-MSC + DHEA contributed to restore ovarian function in aging mice and extend their lifespan by restoring hormone levels and inhibiting inflammatory factors.

## 1. Introduction

In the global population structure transformation and population lifespan extension, there is an inseparable connection between the delay in female reproductive age and the decrease in fertility rate [1]. Therefore, exploring the aging and antiaging of the reproductive system is a research hotspot in the field of reproduction [2, 3, 4]. Ovarian aging is an important indicator of the onset of female aging and causes a decline in endocrine function [5, 6, 7]. The changes in the secretion levels of sex hormones that occur with reproductive aging not only lead to a decline in reproductive function but also are closely related to age-related diseases. Therefore, delaying the aging of the reproductive organs can improve many age-related diseases.

Mesenchymal stem cells (MSCs) in various aged tissues have weakened responses to tissue damage, limited proliferation, and reduced regenerative potential [8, 9]. Studies have shown that MSC treatment for premature ovarian failure has a good effect on ovarian function recovery [10, 11, 12]. However, whether dehydroepiandrosterone (DHEA) can enhance the reversing ovarian natural aging effect of umbilical cord-derived MSCs (UC-MSCs) is still unclear.

The process of ovarian aging is characterized by unique endocrine changes. DHEA is used as a hormone therapy drug in clinical treatment in order to compensate for the decline in endocrine function caused by ovarian aging [13]. Franks and Hardy [14] found a positive correlation between supplementing DHEA and clinical pregnancy rate; Ikeda et al. [15] found that it is not recommended to use DHEA to improve ovarian reserve and reduce induction of ovulation response in women; Gündoğan et al. [16] found that supplementing DHEA can increase the number of transferred embryos and fertilization rate. However, whether DHEA can enhance the reversing ovarian natural aging effect of MSCs is still unclear.

This study used human UC-MSC (hUC-MSC) combined with DHEA to treat aging mice. The aim is to regulate the ovarian microenvironment; restore ovarian function; and delay aging by improving oxidative stress, inflammatory response, and hormone deficiency during the aging process.

## 2. Materials and Methods

### 2.1. Isolation and Culture of hUC-MSC

Human umbilical cord tissue was provided by Beijing Haidian Maternal and Child Health Hospital and was obtained from full-term infants delivered by cesarean section. The umbilical cord was washed with normal saline containing 1% pen-strep solution, and the fascia and arteriovenous vessels were removed. Tissue blocks of 1 mm^3^ were cut, placed in tissue culture dishes, and cultured in a cell incubator at 37°C and 5% CO_2_ used-MEM *α* cell culture medium. After 14 days, the cell growth was observed. When the cells reach about 90% confluence, they are digested with 0.25% trypsin and separated and passaged in a 1 : 2 ratio. hUC-MSCs grown to the fifth generation were prepared for tail vein injection. The fifth-generation hUC-MSCs will be induced for differentiation using the adipogenic differentiation kit (STEMCELL Technologies, VAN, Canada), cartilage differentiation kit (STEMCELL Technologies, VAN, Canada), and osteogenic differentiation kit (STEMCELL Technologies, VAN, Canada), respectively. Use Oil Red O (Applygen Technologies Inc., Beijing, China) staining to identify hUC-MSCs as adipose tissue, Alcian Blue (Applygen Technologies Inc., Beijing, China) staining to identify cartilage, and alkaline phosphatase (Applygen Technologies Inc., Beijing, China) staining to identify bone formation.

### 2.2. Experimental Design

Eighteen-month-old C57BL/6J female mice were purchased from Beijing Huafukang Biotechnology Co., Ltd. The experiment was divided into the control group, hUC-MSC group, DHEA group, and hUC-MSC + DHEA group. Ten mice per group were randomly assigned. The control group was raised normally. Mice in the DHEA group freely drank water containing 0.15 mg/mL DHEA, with a daily intake of about 6–10 mL [17, 18, 19]. Mice in the hUC-MSC group received tail vein transplantation every 14 days 1 × 10^7^/kg hUC-MSC, three transplants in total. The mice in the hUC-MSC + DHEA group were injected in the tail vein every 14 days 1 × 10^7^/kg hUC-MSC and were transplanted three times, and drinking water containing 0.15 mg/mL DHEA was freely consumed daily. All animals underwent neck dissection 60 days after treatment. Observe the life status of mice every day, and record and count the mortality rates of each group. All procedures were approved by the Animal Protection and Use Committee of the National Family Planning Society of China. The use and operation of experimental animals comply with the relevant regulations of the Animal Experiment Management Committee of the Institute of Science and Technology of the National Health Commission.

### 2.3. ELISA Detection of Serum Biochemical Indicators

Mice that fasted for 8 hr were anesthetized with isoflurane inhalation and immediately underwent eye blood collection. Blood samples were placed at 4°C and centrifuged at 4,000 rpm for 10 min to obtain serum. The levels of anti-Mullerian hormone (AMH), estradiol (E2), follicle-stimulating hormone (FSH), malondialdehyde (MDA), superoxide dismutase (SOD), 8-hydroxydeoxyguanosine (8-OHdG), *γ* Interferon (IFN-*γ*), transforming growth factor-*β* (TGF-*β*), tumor necrosis factor-*α* (TNF-*α*), IL-1*β*, IL-6, and IL-18 were measured using Beijing Huabaitai Biotechnology Co., Ltd. Each experiment was repeated three times.

### 2.4. Immunohistochemical Analysis

The expression of Ki67 and p16 in ovary was detected by immunohistochemistry. Rabbit anti-p16 monoclonal antibody (Abcam, Cambridge, UK; 1 : 200) and rabbit anti-Ki67 polyclonal antibody (Abcam, Cambridge, UK; 1 : 200) were mixed with 3% goat serum, and the mixture was added dropwise to ovarian paraffin sections. The nonspecific antibody binding sites were blocked overnight at 4°C. Incubate at room temperature for 1 hr, and add horseradish peroxidase (HRP)-coupled goat anti-rabbit IgG (ZSJQ Bio, Beijing, China, 1 : 1,000). Immunoreactivity was observed using diaminobenzidine (DAB) and hematoxylin staining. Image J software is used to analyze the expression level of immunohistochemistry. Each experiment was repeated three times.

### 2.5. Quantitative Reverse-Transcription Polymerase Chain Reaction (qRT-PCR)

Trizol (Invitrogen, CA, USA) was used to extract ovarian RNA and reverse transcribed into cDNA using a reverse transcription kit (Transgen Biotech, Beijing, China). The qRT-PCR detection kit (SYBR Green, Bio Rad, CA, USA) detects the expression level of the sample. Each experiment was repeated three times. The qRT-PCR primers were designed as follows IL-1*β*-forward, 5'-ATGGGCAACCACTTACCTATTT-3', and IL-1*β*-reverse, 5'-GTTCTAGAGAGTGCTGCCTAATG-3'; IL-6-forward, 5'-TGTTCTCTGGGAAATCGTGG-3', and IL-6-reverse, 5'-CAAGTGCATCATCGTTGTTCATAC-3'; IL-18-forward, 5'-GTTCCCACAACGATGAGTACA-3', and IL-18-reverse, 5'-CTGAGGA TTATAGCAGGCTTCC-3'; AKT-forward, 5'-GTGAGGTTGACAGAGGAACAAG-3', and AKT-reverse, 5'-GCTCTCCTGTCACCAAGATTAAA-3'; mTOR-forward, 5'-GGGAGAACAGAAGATGGGTAAC-3', and mTOR-reverse, 5'-GTTGAGAGGACCAACTGGATTAT-3'; PDK-1-forward, 5'-ACCAACCATCCTGTGAGTTATC-3', and PDK-1-reverse, 5'-CAAGACACCTGGCTACAGTTAT-3'; and PRAS40-forward, 5'-CCCTCCC TAACCCAGAATTG-3'; PRAS40-reverse, 5'-GTCAGGACTCAGGCAAAGAA-3'.

### 2.6. Western Blot Analysis

To test the effect of DHEA on hUC-MSCs, we treated hUC-MSCs with 100 nM DHEA for 24 hr, and the rapamycin group was treated with 20 nM rapamycin. The hUC-MSC protein was extracted by the radio immunoprecipitation assay (RIPA) buffer, quantified with the bicinchoninic acid assay (BCA) protein detection kit (23225, Thermo Fisher Scientific, USA), and separated on 10% polyacrylamide gel. Protein transfer to polyvinylidene fluoride (PVDF) membrane using anti-p-p70 S6K (Abcam, Cambridge, UK; 1 : 500), anti-*β*actin (Abcam, Cambridge, UK; 1 : 1,000), anti-pS6K (Abcam, Cambridge, UK; 1 : 200), anti-pAKT1 (Abcam, Cambridge, UK; 1 : 500), anti-p-mTOR (Biodragon, China; 1 : 200), anti-IL-6 (Abcam, Cambridge, UK; 1 : 200), and anti-TNF-*α* (Abcam, Cambridge, UK; 1 : 200) was incubated overnight at 4°C and bound to goat anti-rabbit IgG conjugated with HRP (ZSJQ Bio, Beijing, China; 1 : 2,000). The protein expression was detected using an enhanced chemiluminescence assay kit (New Cell Molecular Biotech Co., Suzhou, China). Each experiment was repeated three times.

### 2.7. Statistical Analysis

The experimental data were analyzed using SPSS and Prism Graphpad 6.0, represented by the mean ± standard deviation (mean ± SEM). The differences between the two groups of the sample mean were compared using *t*-test. Multiple groups of data were analyzed using one-way ANOVA, with *P* < 0.05 being the significant difference.

## 3. Results

### 3.1. hUC-MSC + DHEA Combined Therapy Improves Ovarian Hypertrophy and Increases Survival Rate

**Figure 1:**
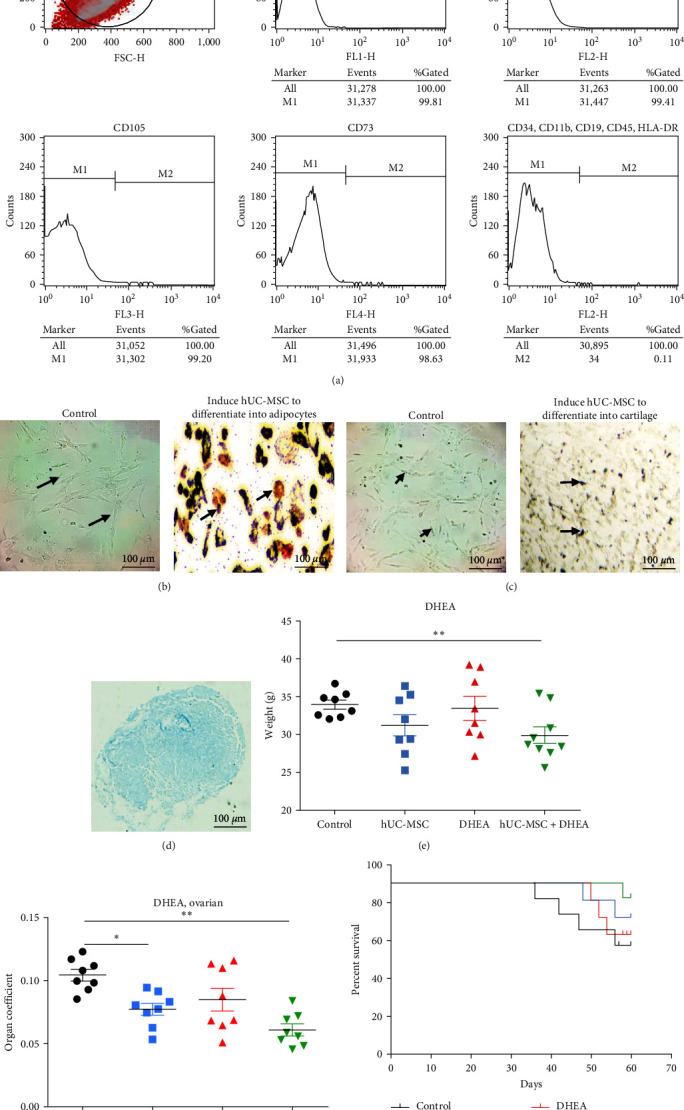
Detection of hUC-MSC cell dryness and mouse natural aging model. (a) Flow cytometry detection of hUC-MSC surface antigen phenotype identification. (b) Induction of adipogenic differentiation in hUC-MSC. (c) Induction of osteogenic differentiation of hUC-MSC. (d) Induction of chondrogenic differentiation in hUC-MSC. (e) 60-day weight testing of aging mice after treatment. (f) Ovarian weight detection in aging mice 60 days after treatment. (g) Mouse survival rate detection.  ^*∗*^*P* < 0.05 and  ^*∗∗*^*P* < 0.01; *n* = 10.

Cell stemness identification was performed before hUC-MSC transplantation was used to treat aging mice. The surface immune antigens of hUC-MSC were detected by flow cytometry; the results showed that the positive rates of CD90, CD44, CD105, and CD73 were greater than 97%; the positive rate of CD34, CD11b, CD19, CD45, and HLA-DR was 0.18% (Figure 1(a)). The differentiation results of hUC-MSC showed that lipid droplets were visible in adipogenic induction (Figure 1(b)), calcium nodules were visible in osteogenic induction (Figure 1(c)), and acidic proteoglycan staining was visible in induced chondrogenesis (Figure 1(d)). These results indicate that the hUC MSCs isolated in this study are stem cells.

Weight gain and organ volume enlargement are common in aging organisms. We conducted statistical analysis on the weight and ovarian weight of mice (Figures 1(d) and 1(f)). The results showed that the weight of mice treated with hUC-MSC + DHEA combination (29.96 ± 1.10) was significantly reduced compared to the control group (34.03 ± 0.60 and *P* < 0.05) and the ovarian weight of the hUC-MSC group (2.40 ± 0.13) and hUC-MSC + DHEA group (1.85 ± 0.13) was significantly reduced compared to the control group (3.63 ± 0.16, *P* < 0.05, and *P* < 0.01). During the treatment period, statistical analysis was conducted on the survival rate of mice, and it was found that compared with the control group, the hUC-MSC group and the hUC-MSC + DHEA combination treatment group showed an improvement in the survival rate of mice (Figure 1(g)). The results suggest that the combined treatment of hUC-MSC + DHEA significantly improves the aging process and ovarian appearance in mice.

### 3.2. hUC-MSC + DHEA Combined Therapy Restores Ovarian Reserve

**Figure 2:**
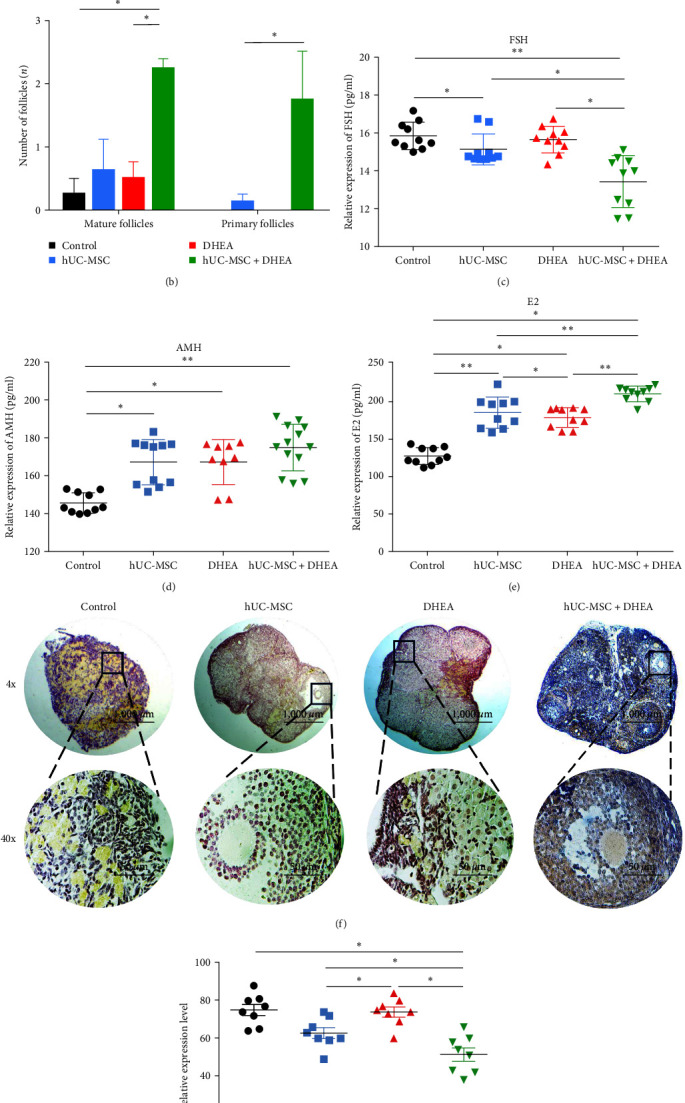
hUC-MSC + DHEA treatment to alleviate aging ovarian function. (a) H&E staining for detecting ovarian structure. Mature follicles have a large cavity and prominent follicles. The primary oocyte is located in the center of the primordial follicle, surrounded by a single layer of flattened follicle cells. Scale bar: 1,000 *μ*m. (b) Statistics on the number of follicles in aging mice after treatment; *n* = 10. (c) ELISA detection of serum FSH expression in mice; *n* = 10. (d) ELISA detection of AMH serum expression in mice; *n* = 10. (e) ELISA detection of serum E2 expression in mice; *n* = 10. (f and g) Immunohistochemical p16 detection of expression levels in ovaries. Scale bar: 1,000 *μ*m; scale bar: 50 *μ*m; *n* = 10.  ^*∗*^*P* < 0.05 and  ^*∗∗*^*P* < 0.01.

Ovarian tissue sections were used to observe the shape of the ovaries and count the number of follicles. The results showed that the control group mainly consisted of corpus luteum and atresia follicles, the number of mature follicles in the hUC-MSC group and DHEA group was increased compared to the control group, and the hUC-MSC + DHEA group not only showed mature follicles but also observed primary follicles (Figures 2(a) and 2(b)). The number of follicles in the hUC-MSC + DHEA group (3.63 ± 0.94) was significantly increased compared to the DHEA (0.50 ± 0.27) and control groups (0.25 ± 0.25, *P* < 0.05, and *P* < 0.01). The results suggest that both hUC-MSC and DHEA can alleviate the decrease in follicle number caused by aging but the combined treatment of DHEA and hUC-MSC can promote the appearance of primary follicles and has a better therapeutic effect on the recovery of follicle number.

### 3.3. hUC-MSC + DHEA Combined Therapy Restores Hormone Levels

Enzyme-linked immunosorbent assay (ELISA) detection of serum hormone levels in aging mice was used to evaluate the recovery of ovarian function (Figures 2(c), 2(d), and 2(e)). The results showed that compared with the control group (15.88 ± 0.23, 145.8 ± 1.72, and 126.8 ± 3.54), the expression levels of FSH were significantly reduced in the hUC-MSC group (15.16 ± 0.26, 167.5 ± 3.622, and 184.5 ± 6.60) and the hUC-MSC + DHEA group (13.45 ± 0.43, 175.2 ± 3.43, and 209.3 ± 3.14), while the expression levels of AMH and E2 were significantly increased (*P* < 0.05 and *P* < 0.01). Among them, there were significant differences in the expression levels of FSH and E2 between the hUC-MSC group and the hUC-MSC + DHEA group (*P* < 0.05). There were significant differences in the expression levels of AMH and E2 between the control group and the DHEA group (167.6 ± 3.97, 177.9 ± 4.13, and *P* < 0.05). The results indicate that both hUC-MSC transplantation and the combined therapy of hUC-MSC + DHEA can alleviate the phenomenon of decreased AMH and E2, as well as increased FSH caused by aging. The combined treatment of hUC-MSC + DHEA has a more remarkable effect on the recovery of ovarian function.

### 3.4. hUC-MSC + DHEA Combined Therapy Alleviates Ovarian Aging

**Figure 3:**
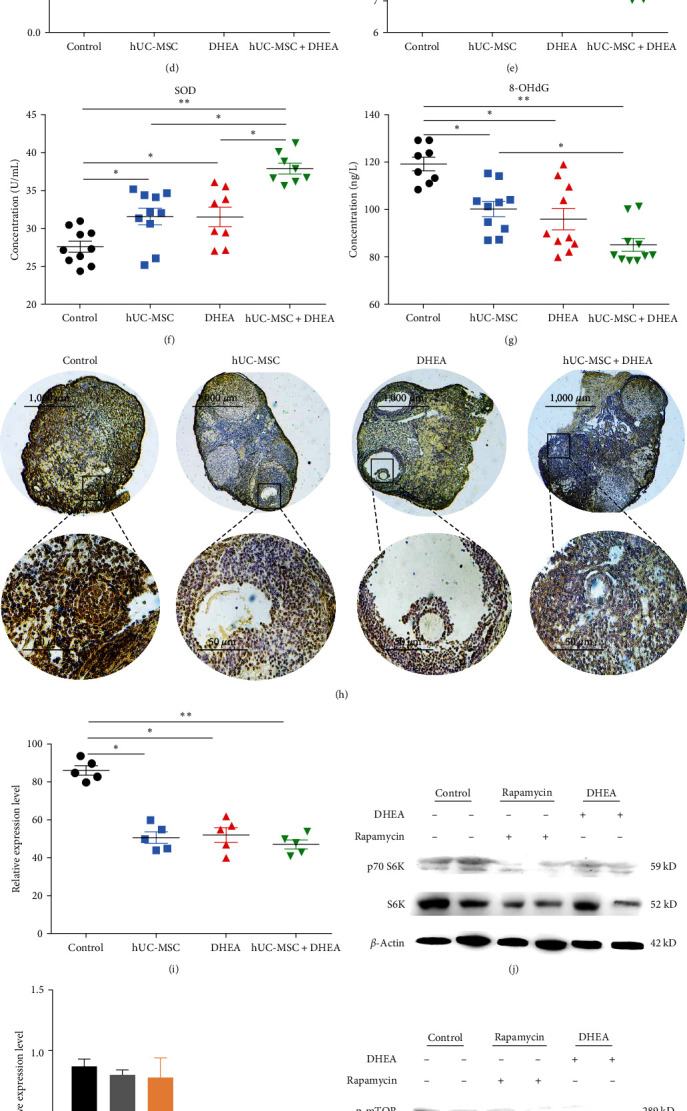
hUC-MSC + DHEA treatment inhibits ovarian aging and reduces oxidative stress levels. (a and b) Immunohistochemical Ki67 detection of expression levels in ovaries. Scale bar: 1,000 *μ*m; scale bar: 50 *μ*m; *n* = 10. (c) Detection of p16 expression in ovaries by qRT-PCR; *n* > 7. (d) Detection of p21 expression in ovaries by qRT-PCR; *n* > 8. (e) ELISA detection of serum MDA expression in mice; *n* = 10. (f) ELISA detection of serum SOD expression in mice; *n* = 10. (g) ELISA detection of serum 8-OHdG expression in mice; *n* > 8. (h and i) Immunohistochemical 8-OHdG detection of expression levels in ovaries. (j and k) p70 S6K and S6K expression in ovaries tissue using western blot. (l and m) p-mTOR, p-AKT1, and IL-6 expression in ovaries tissue using western blot. (n and o) p-PI3K and TNF-*α* expression in ovary tissues using western blot. Scale bar: 1,000 *μ*m; scale bar: 50 *μ*m; *n* = 10.  ^*∗*^*P* < 0.05 and  ^*∗∗*^*P* < 0.01.

To evaluate the effect of treatment on aging mouse ovaries, we detected the expression of aging marker p16 in the ovaries through immunohistochemistry (Figures 2(f) and 2(g)). The results showed that the hUC-MSC + DHEA group (51.50 ± 3.47) was significantly reduced compared to the control group (75.13 ± 2.88) and hUC-MSC group (62.88 ± 2.81), while the DHEA group (74.00 ± 2.563) was significantly increased compared to the hUC-MSC group and hUC-MSC + DHEA group (*P* < 0.05 and *P* < 0.01). The RT-PCR detection of the expression levels of p16 and p21 in the ovaries showed consistent results (Figures 3(c) and (d)). Compared with the control group (1.00 ± 0.00 and 1.00 ± 0.00), the expression levels of p16 and p21 were significantly reduced in the hUC-MSC group (0.82 ± 0.06 and 0.77 ± 0.03) and hUC-MSC + DHEA group (0.48 ± 0.05 and 0.53 ± 0.03), and significant differences were also observed between these two groups (*P* < 0.05 and *P* < 0.01). We also detected the expression level of Ki67 in the ovaries (Figures 3(a) and 3(b)), and the results showed that the expression level of hUC-MSC + DHEA group (55.00 ± 2.68) was significantly increased compared to the control group (42.50 ± 2.72). Compared with the DHEA group (41.63 ± 1.69), the expression levels of the hUC-MSC group (55.50 ± 2.08) and hUC-MSC + DHEA group were significantly increased (*P* < 0.05 and *P* < 0.01). The results showed that the combination therapy of hUC-MSC and DHEA effectively slowed down ovarian aging.

### 3.5. hUC-MSC + DHEA Combined Therapy Alleviates Oxidative Stress Levels

Oxidative stress levels in mice were determined by measuring the expression levels of MDA, SOD, and 8-OHdG (Figures 3(e), 3(f), and 3(g)). The results showed that compared with the control group (10.42 ± 0.18, 119.5 ± 2.90, and 27.63 ± 0.73), the expression levels of MDA and 8-OHdG were significantly reduced in the hUC-MSC group (8.66 ± 0.46, 100.4 ± 3.18, and 31.63 ± 1.11), DHEA group (9.45 ± 0.13, 96.11 ± 4.58, and 31.57 ± 1.29), and hUC-MSC + DHEA group (7.72 ± 0.35, 85.21 ± 2.76, and 38.00 ± 0.72), while the expression levels of SOD were significantly increased (*P* < 0.05 and *P* < 0.01). The expression level of 8-OHdG in the hUC-MSC + DHEA group was significantly reduced compared to the hUC-MSC group (*P* < 0.05), while the expression level of SOD was significantly increased (*P* < 0.05). The results suggest that hUC-MSC + DHEA exerts antioxidant effects, and the combined treatment of hUC-MSC + DHEA has a better effect on alleviating oxidative stress levels and DNA damage caused by aging in the body.

We detected the expression of 8-OHdG in mouse ovaries (Figures 3(h) and 3(i)). The results showed that compared with the control group (86.40 ± 2.502), the oxidative activity of the ovaries in the DHEA group (52.20 ± 3.891 and *P* < 0.05), hUC-MSC group (50.80 ± 3.023 and *P* < 0.05), and hUC-MSC + DHEA group (47.20 ± 2.354 and *P* < 0.01) was significantly reduced. Among them, the hUC-MSC + DHEA group showed a more significant decrease. The results indicate that treatment reduced the level of DNA damage in mouse ovaries.

### 3.6. hUC-MSC + DHEA Combined Therapy Inhibits Inflammatory Factors

The effects of DHEA treatment on the PI3K/AKT/mTOR pathway and inflammatory factors in hUC-MSC were detected (Figures 3(j), 3(k), 3(l), 3(m), 3(n), and 3(o)). The expressions of p-mTOR and p70 S6K in the rapamycin group (0.055 ± 0.01 and 0.045 ± 0.01) and DHEA group (0.017 ± 0.00, 0.015 ± 0.01) were significantly lower than those in the control group (0.23 ± 0.03, 0.19 ± 0.04, and *P* < 0.05), and the expression levels of IL-6 in the rapamycin group (0.65 ± 0.05) were significantly increased compared to the control group (0.25 ± 0.05) and DHEA group (0.04 ± 0.02 and *P* < 0.05), but the difference in TNF-*α* expression was not significant. At the same time, the expression levels of p-AKT1 and p-PI3K were not significantly different among the groups. The results suggest that there may be no reciprocal regulation between PI3K/Akt/mTOR pathway activation and proinflammatory cytokine inhibition.

**Figure 4:**
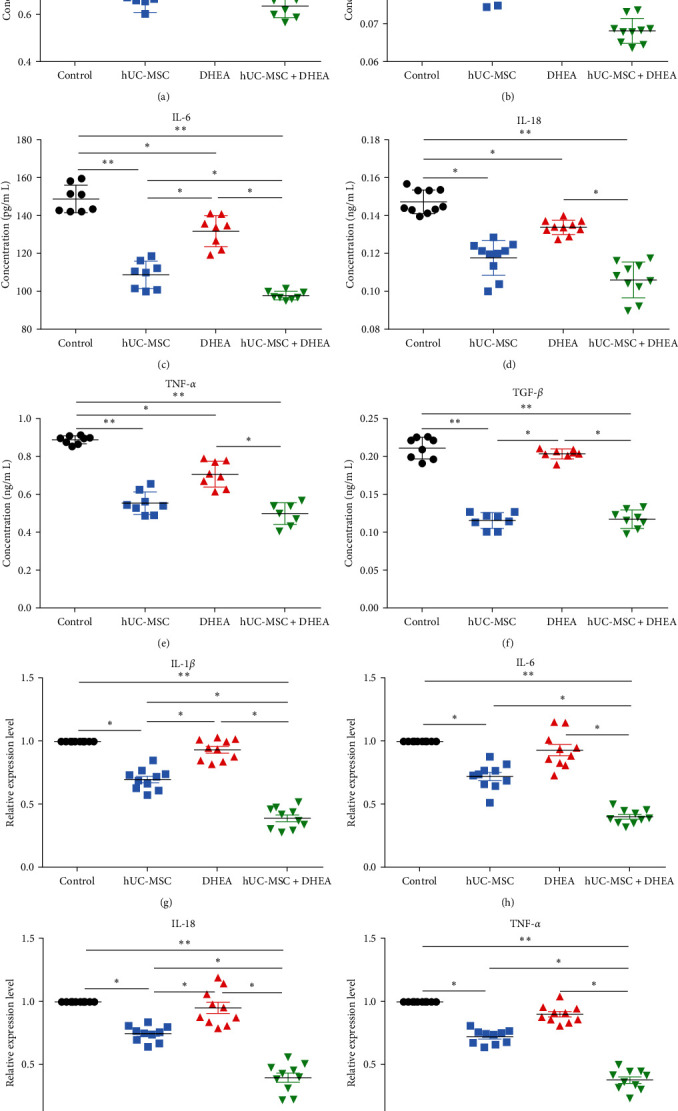
hUC-MSC + DHEA treatment inhibits inflammation in aging mice. ELISA detection in mice of (a) serum IFN-*γ* expression; *n* = 8. (b) Serum IL-1*β* expression; *n* = 8. (c) Serum IL-6 expression; *n* = 8. (d) Serum IL-18 expression; *n* = 8. (e) TNF-*α* expression; *n* = 8. (f) Serum TGF-*β* expression; *n* = 8. (g) Detection of IL-1*β* expression in ovaries by qRT-PCR. (h) Detection of IL-6 expression in ovaries by qRT-PCR. (i) Detection of IL-18 expression in ovaries by qRT-PCR. (j) Detection of TNF-*α* expression in ovaries by qRT-PCR; *n* > 7;  ^*∗*^*P* < 0.05 and  ^*∗∗*^*P* < 0.01.

The accumulation of chronic inflammation promotes aging. Therefore, we detected the expression of proinflammatory factors IFN-*γ*, IL-1 *β*, IL-6, IL-18, TNF-*α*, and TGF-*β* in the serum and ovaries of aging mice (Figures 4(a), 4(b), 4(c), 4(d), 4(e), 4(f), 4(g), 4(i), and 4(j)). The results showed that compared with the control group (0.95 ± 0.01, 0.10 ± 0.00, 149.2 ± 2.59, 0.15 ± 0.00, 0.89 ± 0.01, and 0.21 ± 0.01), the expression of IFN-*γ*, IL-1*β*, IL-6, IL-18, TNF-*α*, and TGF-*β* in the serum of the DHEA group (0.86 ± 0.03, 0.09 ± 0.00, 132.0 ± 2.92, 0.13 ± 0.00, 0.71 ± 0.02, and 0.20 ± 0.00), hUC-MSC group (0.71 ± 0.04, 0.08 ± 0.00, 108.9 ± 2.56, 0.12 ± 0.00, 0.56 ± 0.02, and 0.12 ± 0.00), and hUC-MSC + DHEA group (0.64 ± 0.02, 0.07 ± 0.00, 97.88 ± 0.79, 0.11 ± 0.00, 0.50 ± 0.02, and 0.12 ± 0.00) was reduced. Among them, the expression levels of IL-6 and IL-1*β* were significantly reduced in the hUC-MSC + DHEA group compared to the hUC-MSC group (*P* < 0.05). In ovarian tissue, the expression levels of IL-1*β*, IL-6, IL-18, and TNF-*α* decreased in the DHEA group (0.93 ± 0.03, 0.93 ± 0.04, 0.95 ± 0.04, and 0.90 ± 0.02), hUC-MSC group (0.70 ± 0.03, 0.72 ± 0.03, 0.75 ± 0.02, and 0.72 ± 0.02), and hUC-MSC + DHEA group (0.39 ± 0.03, 0.46 ± 0.02, 0.40 ± 0.04, and 0.38 ± 0.03) compared to the control group (1.00 ± 0.00, 1.00 ± 0.00, 1.00 ± 0.00, and 1.00 ± 0.00). The expression of proinflammatory factors in the hUC-MSC + DHEA group was significantly reduced compared to the hUC-MSC group (*P* < 0.05). The results suggest that the combination therapy of hUC-MSC + DHEA may inhibit the expression of proinflammatory factors in the ovaries and serum by regulating the ovarian microenvironment and alleviating ovarian aging.

### 3.7. hUC-MSC + DHEA Combined Therapy Inhibits the PI3K/AKT/mTOR Pathway

**Figure 5:**
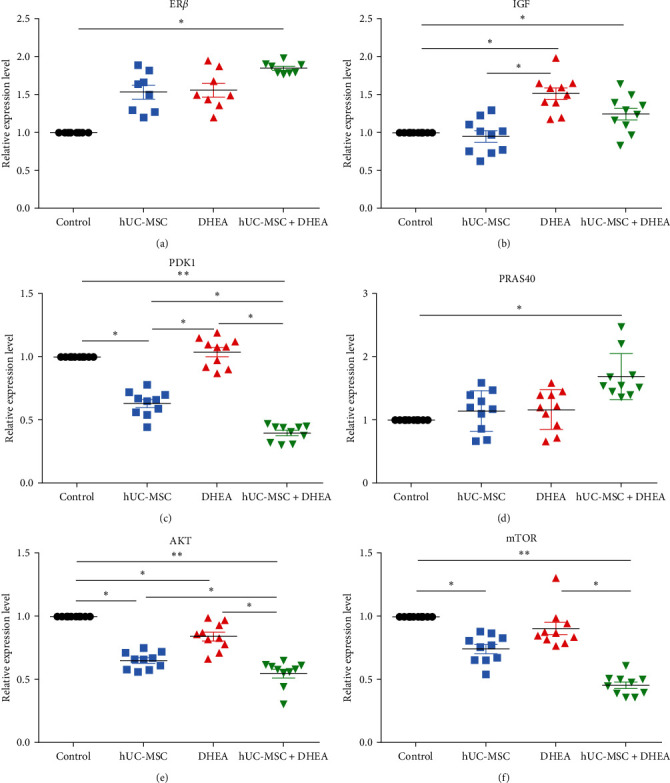
hUC-MSC + DHEA combined therapy inhibits the PI3K/AKT/mTOR pathway. Detection of (a) ER*β* expression in uterine using qRT-PCR; *n* = 10. (b) IGF expression in ovaries by qRT-PCR; *n* = 10. (c) PDK1 expression in ovaries by qRT-PCR; *n* = 10. (d) PRAS40 expression in ovaries by qRT-PCR; *n* = 10. (e) AKT expression in ovaries by qRT-PCR; *n* = 10. (f) mTOR expression in ovaries by qRT-PCR; *n* = 10.  ^*∗*^*P* < 0.05 and  ^*∗∗*^*P* < 0.01.

The PI3K/AKT/mTOR pathway is a classic pathway that regulates aging. To explore the molecular mechanism of hUC-MSC + DHEA inhibiting aging, qRT-PCR was used to detect the expression of genes related to the PI3K/AKT/mTOR pathway in the ovaries. The results showed that, compared to the control group, the hUC-MSC + DHEA group significantly increased the expression levels of ER*β* (1.85 ± 0.03), IGF (1.25 ± 0.08), and PRAS40 (1.69 ± 0.12), while the expression levels of PDK1 (0.39 ± 0.02), AKT (0.55 ± 0.03), and mTOR (0.46 ± 0.03) were significantly reduced (*P* < 0.05 and *P* < 0.01; Figures 5(a), 5(b), 5(c), 5(d), 5(e), and 5(f)). IGF and ER*β* can activate the PI3K/AKT pathway, and PRAS40 expression may activate TNF-*α*-induced mTORC1 signaling [20], suggesting that hUC-MSC + DHEA may inhibit aging through the PI3K/AKT/mTOR pathway.

## 4. Discussion

Aging is a time-dependent physiological deterioration that is a complex process involving numerous molecular mechanisms. During the aging process, significant alterations have also occurred in endocrine function [21, 22, 23, 24]. DHEA is a steroid mainly produced by the adrenal reticular zone and can also be synthesized in small quantities in the human ovary. Its secretion decreases with age [25, 26, 27]. This has aroused research interest in its utilization as an “antiaging” hormone. Although DHEA is an androgen, it is an intermediate steroid in the biosynthesis pathway of testosterone and estradiol, which can be transformed into estrogen and androgens to exert its function [28, 29, 30, 31, 32]. Casson et al. [31] found that DHEA supplementation may maximize ovarian response in patients with poor ovarian response. There are also research reports that among women with reduced ovarian reserve, different doses of DHEA are administered, and some of them have improved pregnancy rates [33, 34, 35, 36, 37]. Research has found that transplanting the ovaries of young mice to older mice does not restore their fertility [38, 39]. This suggests that the internal environment of the organism is crucial for the functioning of the ovaries.

The therapeutic effect of stem cells is mediated through homing, differentiation, and paracrine activation [40]. Long-term chronic inflammation can accelerate the aging process within the body, and hUC-MSC possesses excellent anti-inflammatory effects [41, 42, 43]. Therefore, we used a combination of hUC-MSC + DHEA to improve the ovarian microenvironment of aging mice and detect the changes in the tissue structure and function of the aging ovaries. The most important manifestation of ovarian aging is a reduction in the quantity and quality of follicles, and there is abundant evidence suggesting that DHEA promotes the growth of preantral and antral follicles [44, 45]. Through H&E staining, we observed that the combination of hUC-MSC + DHEA treatment in 18-month-old mice significantly restored the number of follicles, particularly primary follicles, after 2 months of treatment. The ovary is the first organ to age in women [46, 47]. Supplementing with DHEA or hUC-MSC had effects on weight loss, ovarian weight reduction, and prolonging the lifespan of aging mice. In addition, hUC-MSC + DHEA had a more significant therapeutic effect on ovarian aging. This indicates that the combined treatment of hUC-MSC + DHEA has a significantly enhanced effect on inhibiting aging compared to individual treatment. The unique feature of the ovarian aging process is endocrine changes [48, 49, 50], and we detected the expression levels of AMH, FSH, and E2. The combined treatment of hUC-MSC + DHEA significantly upregulated the expression of E2 and AMH in the serum of aging mice compared to the control group, while the expression of FSH was significantly downregulated, signifying a significant improvement in ovarian function compared to the control group. At the same time, we found that compared with the hUC-MSC group, the hUC-MSC + DHEA group exhibited higher hormone levels (FSH and E2), which might promote the functional recovery of the ovary. Immunohistochemical staining of ovarian tissue revealed that specific staining was mainly concentrated in ovarian stromal cells in the control group and DHEA group. Specific staining was observed in mature oocyte granulosa cells in the hUC-MSC group, while less specific staining was observed in mature oocyte granulosa cells in the hUC-MSC + DHEA group. Compared with the hUC-MSC group, the number of mature follicles and primordial follicles in the hUC-MSC + DHEA group was significantly increased. To further validate the results, we detected the expression levels of aging markers p16 and p21 in the ovaries and conducted immunohistochemical detection of p16 and Ki67 expression in the ovaries. It was discovered that hUC-MSC + DHEA significantly inhibited ovarian aging and cell proliferation recovery was observed in ovarian granulosa cells and the ovarian matrix.

To explore the role of inflammation in improving ovarian function, we investigated the proinflammatory factors IL-1, IL-6, IL-18, IFN-*γ*, and TNF-*α* through testing; it was discovered that hUC-MSC + DHEA might improve ovarian function by suppressing proinflammatory factors. Simultaneously, we found that compared with the hUC-MSC group, the hUC-MSC + DHEA combined treatment group had a better inhibitory effect on inflammation. At the same time, we observed that the expression level of the classic aging-related pathway PI3K/AKT/mTOR was significantly reduced in the hUC-MSC + DHEA combination treatment group compared to the control group. However, conversely, a decrease in proinflammatory cytokines may not necessarily imply that the PI3K/AKT/mTOR pathway will definitely be activated. For example, nintedanib treating idiopathic pulmonary fibrosis can reduce the expression of proinflammatory factors IL-1*β*, IL-6, and TNF-*ɑ* by downregulating the PI3K/Akt/mTOR pathway [51]. In lipopolysaccharide (LPS)-induced macrophages, scoparone could suppress the expression of proinflammatory factors IL-6 and TNF-*α* by inhibiting the PI3K/AKT/mTOR pathway [52]. The MSC-derived extracellular vesicles (MSC-EVs) treatment significantly inhibited the upregulation of IL-6, IL-1*β*, and TNF-*α* and the activation of the PI3K/AKT/mTOR signaling pathway in LPS-stimulated microglia [20]. These research findings are similar to ours. From these results, it can be seen that there may not be a mutually regulatory relationship between PI3K/Akt/mTOR pathway activation and proinflammatory cytokine inhibition. Ling et al. [53] found that MSC secretion of IGF can improve ovarian function and DHEA can regulate hormone levels in the body. Our experimental results also found that the expression levels of FSH and AMH were upregulated in the DHEA group compared to the hUC-MSC group, thereby activating the PI3K/AKT pathway. These results suggest that the combination therapy of hUC-MSC and DHEA has a wider impact on ovarian function-related pathways compared to hUC-MSC or DHEA alone, achieving better therapeutic effects. These results suggest that the combination of hUC-MSC + DHEA significantly improves the ovarian microenvironment, providing ideas for finding methods to alleviate ovarian aging in the future.

## Data Availability

The datasets during and/or analyzed during the current study are available from the corresponding author on reasonable request.
